# Multivitamins and minerals modulate whole-body energy metabolism and cerebral blood-flow during cognitive task performance: a double-blind, randomised, placebo-controlled trial

**DOI:** 10.1186/s12986-016-0071-4

**Published:** 2016-02-11

**Authors:** David O. Kennedy, Emma J. Stevenson, Philippa A. Jackson, Sarah Dunn, Karl Wishart, Gregor Bieri, Luca Barella, Alexandra Carne, Fiona L. Dodd, Bernadette C. Robertson, Joanne Forster, Crystal F. Haskell-Ramsay

**Affiliations:** Brain, Performance and Nutrition Research Centre, Northumbria University, Newcastle-upon-Tyne, NE1 8ST UK; Bayer HealthCare – Consumer Care, Peter Merian Strasse 84, P.O. Box 4002, Basel, Switzerland

**Keywords:** Cognitive, Metabolism, Indirect calorimetry, NIRS, Cerebral blood-flow, Vitamins, Minerals, Coenzyme Q10

## Abstract

**Background:**

The brain is by far the most metabolically active organ in the body, with overall energy expenditure and local blood-supply closely related to neural activity. Both energy metabolism and cerebral vaso-dilation are dependent on adequate micronutrient status. This study investigated whether supplementation with ascending doses of multi-vitamin/minerals could modulate the metabolic and cerebral blood-flow consequences of performing cognitive tasks that varied in difficulty.

**Methods:**

In this randomised, double-blind, placebo-controlled, parallel-groups study 97 healthy females (25–49 y), who were not selected on the basis of any nutritional parameters, received either placebo or one of two doses of multivitamins/minerals. Cerebral blood-flow (CBF) parameters in the frontal cortex, and total energy expenditure (TotalEnergy), carbohydrate and fat oxidation (CarbOxi/FatOxi), were measured during 5 tasks of graded cognitive difficulty and a control task (5 min per task) using Near-infrared spectroscopy (NIRS) and Indirect calorimetry of exhaled pulmonary gas (ICa) respectively. Assessments took place 60 min after the first dose and following eight weeks supplementation.

**Results:**

During task performance supplementation with the first dose of micronutrients led to a dose-dependent increase in TotalEnergy and FatOxi throughout the post-dose assessment period following the higher dose, and increases in the total concentration of haemoglobin, a proxy measure for CBF, during task performance following the lower dose of vitamins/minerals (also containing coenzyme-Q10). Chronic supplementation over 8 weeks led to a dose-dependent increase in TotalEnergy during the task period. There were no interpretable effects on mood or cognitive performance.

**Conclusions:**

These results show that acute supplementation with micronutrients in healthy adults can modulate metabolic parameters and cerebral blood flow during cognitive task performance, and that the metabolic consequences are sustained during chronic supplementation. These findings suggest that both brain function and metabolism are amenable to micronutrient supplementation, even in adults who are assumed to have nutritional status typical of the population.

**Trial registration:**

ClinicalTrials.gov - NCT02381964.

**Electronic supplementary material:**

The online version of this article (doi:10.1186/s12986-016-0071-4) contains supplementary material, which is available to authorized users.

## Background

Whilst the brain represents only approximately 2 % of body weight, it receives 11 % of cardiac output and accounts for more than 20 % of the body’s total energy expenditure [1]. Most of the brain’s energy requirement is expended on neural signalling, with the high baseline levels of energy expenditure during resting wakefulness being augmented by comparatively small increases during focussed activity [1, 2]. During these periods of neural activity metabolic substrates (glucose/oxygen) cross the blood brain barrier and are delivered, on demand, to active brain areas via dilation of the local vasculature. Nitric oxide is a key mediator of this ‘neurovascular coupling’ between neuronal activity and increased blood supply in active tissue [3, 4] and is released from cortical neurons in an activity-dependent manner [5]. The direct local increases in blood flow or metabolic substrates during local activity provide the proxy markers of activity for brain imaging techniques such as functional magnetic resonance imaging (fMRI) and positron emission tomography (PET). Interestingly, several studies have used indirect calorimetry (ICa) of exhaled pulmonary air to investigate changes in whole-body metabolism during brain activity. These studies have demonstrated increased overall energy expenditure during the performance of cognitive tasks [6] and during rapid eye movement (REM) sleep, as compared to other sleep stages [7], and a shift towards fat oxidation during the middle and later stages of a chess game [8].

Vitamins and minerals play both direct roles in brain function (e.g. via neurotransmitter synthesis, receptor binding, membrane ion pump function) as well as indirect roles, for instance via their involvement in energy metabolism and the modulation of cerebral blood supply [9, 10]. With regards cellular energy production, most of the 13 vitamins and a number of minerals play direct or indirect roles in mitochondrial function [10–12]. As an example, the water-soluble B vitamins play ubiquitous and essential interacting roles as coenzymes and precursors in the majority of cellular functions. Of particular relevance here vitamins B_1_, B_2_, B_3_, B_5_ are essential co-factors in mitochondrial aerobic respiration and cellular energy production via their roles in the tricarboxylic acid cycle, the electron transport chain and the formation of adenosine triphosphate (ATP), the cell’s energy currency; whereas vitamins B_6_, B_9_, and B_12_ play essential roles in all aspects of one-carbon metabolism [10, 12–14]. Similarly, coenzyme Q10 (CoQ10) is an essential coenzyme required alongside the vitamins for the mitochondrial production of ATP via oxidative phosphorylation. CoQ10, which is also a potent antioxidant, is synthesised endogenously at a low rate, and its level can be augmented from dietary sources. It has been noted that deficiencies in both the B vitamins and CoQ10 are associated with fatigue and a number of conditions related to compromised mitochondrial function [10, 13, 15]. The role of multiple vitamins in overall somatic energy production has been investigated directly in a single study conducted in 87 obese females which used ICa to assess the effects of 26 weeks supplementation with multi-vitamins/minerals or placebo on energy expenditure. The results showed that supplementation was associated with a significant increase in resting energy expenditure and fat oxidation [16].

As well as pivotal roles in mitochondrial energy production, both vitamins and CoQ10 are also associated with cardiovascular functioning, and therefore, by implication, the delivery of metabolic substrates to the brain. As an example, the status of vitamin C [17], vitamin D [18] and B vitamins [19] have all been shown to be related to peripheral blood flow as indicated by measures of endothelial function. Similarly, single doses of vitamins B_9_, C, E and C/E combined have all been shown to increase vasodilation, as measured by flow mediated dilation, in groups with disease or dietary manipulation related endothelial dysfunction [20–23]. Similarly, there is evidence that longer term supplementation with vitamin C [24–26], vitamin B_12_ [27], vitamin B_9_ [28] and vitamin D [29–31] can improve endothelial function in groups with poor endothelial function, cardio-metabolic disorders or a deficiency in the specific vitamin. In the case of CoQ10, a recent meta-analysis of the results of five methodologically adequate studies [32] suggested that supplementation with CoQ10 can also engender improvements in endothelial function and other cardiovascular parameters. The underlying hypothesis for the vasodilatory effects of these micronutrients typically relates to their antioxidant properties. However, in the case of vitamin C [24, 25], vitamin B_9_ [28]. and CoQ10 [32], specific roles in the synthesis of nitric oxide or the activity of nitric oxide synthase have been identified.

The relationship between micronutrients and energy production/blood flow becomes most pertinent in light of evidence suggesting that a substantial proportion of the general population of developed countries are deficient in one or more vitamins and minerals [12, 33, 34]. The reversal of marginal insufficiencies/deficiencies may well underlie recent demonstrations of improved mood or cognitive performance following supplementation with multi-vitamins/minerals in healthy cohorts of children [35, 36] and adults [37, 38]. Given the above, the current study investigated whether supplementation with multi-vitamins/minerals (± CoQ10) could modulate the increases in energy expenditure and concentrations of haemoglobin, a proxy measure of local cerebral blood flow (CBF), associated with differing intensities of cognitive tasks, using ICa and near-infrared spectroscopy (NIRS) respectively. This randomised, double-blind, placebo-controlled, parallel-groups study therefore involved the monitoring of metabolic parameters and CBF parameters during five tasks that exerted varying levels of cognitive demand and a somatically matched control task.

## Methods

### Design

The study employed a randomised, double-blind, placebo-controlled, parallel groups design with energy metabolism, and cerebral hemodynamics during cognitive tasks being measured following a single dose (Day 1) and after eight weeks (Day 56) supplementation with one of two multivitamin/mineral supplements or placebo. In this exploratory study both the metabolic and CBF parameters were considered primary outcomes and all other measures secondary outcomes.

### Treatments

The three treatment groups comprised:

- Supradyn® 1RDA + CoQ10, containing vitamins/minerals at levels up to 100 % of the 2008 European Union recommended dietary allowances (RDA), plus 4.5 mg CoQ10 (1RDA + Q10).
- Supradyn® 3RDA, containing vitamins and minerals at levels up to 300 % (water soluble vitamins only) of the 1990 European Union RDAs (3RDA).
- Placebo matched for appearance, taste, and odour (composed of excipients from the active formulae: talc, magnesium stearate, microcrystalline cellulose).

The active constituents of the multivitamin/mineral interventions are shown in Table 1.

**Table 1:** Composition of the 1RDA + Q10 and 3RDA interventions

Active	Units	1RDA^1^ +CoQ10	3RDA^2^
Vitamin A	μg	800	800
Vitamin B1	mg	1.1	4.2
Vitamin B2	mg	1.4	4.8
Vitamin B6	mg	1.4	6
Vitamin B12	μg	2.5	3
Vitamin C	mg	80	180
Vitamin D3	μg	5	5
Vitamin E	mg	12	10
Vitamin K1	μg	25	30
Biotin	μg	50	450
Folic acid	μg	200	600
Niacin	mg	16	54
Pantothenic acid	mg	6	18
Calcium	mg	120	120
Copper	mg	1	0.9
Iodine	μg	150	75
Iron	mg	14	8
Magnesium	mg	80	45
Manganese	mg	2	1.8
Molybdenum	μg	50	45
Selenium	μg	50	55
Zinc	mg	10	8
Coenzyme Q10	mg	4.5	
Phosphorus	mg		126.3
Chromium	μg		25
Fluoride	mg		1.5

^1^Up to 1 RDA as per EU directive 2008/100/EC (2008) ^2^ Up to 3 RDAs as per EU directive 90/496/EEC (1990)

The interventions were provided in a single bottle containing film coated tablets sufficient for a 63 day supplementation period. All treatments were prepared and bottled by the manufacturer in accordance with a computer-generated randomisation list and delivered to the investigational site identified only by their randomisation code. Participants were allocated sequentially to the randomisation code list. One dose of the intervention was taken orally, with water, once a day over a period of 56 days. Compliance was assessed with treatment diaries, pill counting, and an interim (~Day 28) phone conversation.

### Participants

**Fig. 1:**
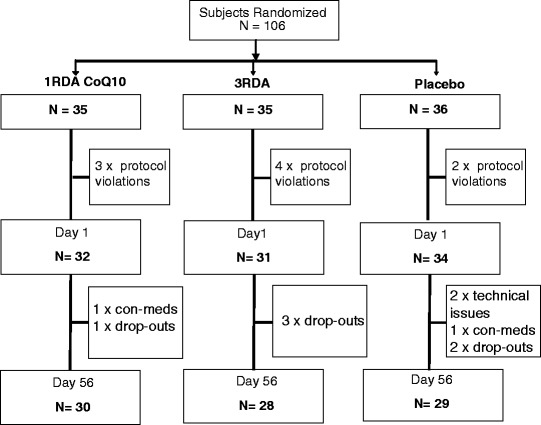
Subject disposition flowchart

A total of 106 females in the age range 25 to 49 years were randomised. Following drop-outs and exclusions, as identified during a blind data-review meeting, a total of 87 participants who had provided a full set of data on Day and Day 56 were included in the statistical analyses. Participant demographics and dispositions are shown in Table 2/Fig. 1.

**Table 2:** Group demographics, including the results of one-way ANOVAs confirming equivalence on all parameters

	1RDA + Q10 *N* = 32		3RDA *N* = 31		Placebo *N* = 34			
Task period	Mean	SEM	Mean	SEM	Mean	SEM	F	p
Age (years)	32.97	1.26	33.65	1.26	33.38	1.32	0.07	0.93
Height (cm)	166.09	0.92	166.13	1.15	165.24	1.17	0.22	0.80
Weight (kg)	66.08	1.70	67.55	1.96	64.39	1.40	0.88	0.42
BMI (kg/m^2^)	23.94	0.57	24.55	0.77	23.64	0.55	0.53	0.59
Fruit and veg (portions per day)	3.98	0.24	3.87	0.27	4.62	0.29	2.24	0.11
Physical activity (hours per week)	3.28	0.53	3.42	0.70	3.79	0.47	0.22	0.80
Weekly alcohol consumption (units)	6.5	0.81	5.29	0.82	6.47	0.98	0.6	0.55
Daily caffeine consumption (mg)	158.42	20.51	167.59	20.92	169.21	19.14	0.08	0.92
Systolic blood pressure (mm/Hg)	114.47	1.57	117.55	1.74	116.47	1.53	0.92	0.40
Diastolic blood pressure (mm/Hg)	78.16	1.38	77.16	1.57	78.15	1.23	0.17	0.85
Heart rate (bpm)	68.63	1.87	66.16	1.95	69.56	2.09	0.78	0.46
Respiratory rate	15.44	0.51	14.55	0.66	14.38	0.50	1.03	0.36
Tympanic temperature	36.82	0.07	36.93	0.05	36.87	0.07	0.66	0.52

All participants had a body mass index (BMI) in the range of 18.50-34.99 kg/m^2^ and they did not exhibit any clinically relevant deviations from normal blood pressure, pulse rate, respiratory rate and body temperature. All participants also self-reported that they were healthy, did not currently take any pharmaceutical treatments (with exception of oral contraceptives, or other routine medications to treat benign conditions) and did not have a history or current diagnosis of any relevant disease, including neurological or psychiatric diseases, significant head trauma, migraines within the last five years, or drug/alcohol abuse. Participants also self-reported that they were not pregnant or lactating and, if relevant, were using a medically acceptable form of birth control; that they did not smoke or consume excessive daily levels of caffeine (>500 mg caffeine per day); that they had not taken significant quantities of dietary supplements within the last 4 weeks. Participants agreed to abstain from alcohol for 24 h and to fast (with the exception of water) for 12 h before each visit, and to abstain throughout the trial from intake of any herbal extracts or dietary supplements. The participants’ continued compliance with the inclusion/exclusion criteria was confirmed at the start of each visit to the laboratory.

This study was conducted according to the guidelines laid down in the Declaration of Helsinki and all procedures involving human subjects/patients were approved by the Northumbria University Faculty of Health and Life Sciences ethics committee. Written informed consent was obtained from all subjects. The trial was registered on ClinicalTrials.gov - Identifier: NCT02381964.

### Measures

#### Energy metabolism - indirect calorimetry (ICa)

Oxygen uptake and carbon dioxide production were measured from expired pulmonary air using an on-line gas analysis system (Metalyzer 3B, Cortex, Leipzig, Germany). These data were used to calculate total energy expenditure (TotalEnergy), and fat and carbohydrate metabolism (FatOxi and CarbOxi respectively) using the standard formulae [39]. During the sampling period subjects breathed into a mask covering the nose and mouth; this was connected to the Metalyzer via falconia tubing. All subjects were familiarised with the procedure before commencing the trial. The Metalyzer 3B also collected heart rate data.

#### Cerebral blood-flow parameters - near- infrared spectroscopy (NIRS)

NIRS is non-invasive brain imaging technique predicated on the absorption by oxygenated (Oxy-Hb) and deoxygenated (Deoxy-Hb) hemoglobin of differing wavelengths of infra-red light, introduced through the intact scalp/skull. Continuous-wave NIRS (CW-NIRS) can be used to assess acute changes in local CBF, as indexed by concentration changes in total hemoglobin during a single continuous recording session. Given that CW-NIRS generates concentration change data that is intrinsically baseline-adjusted to the concentration immediately prior to the first data point in the recording session, it cannot be used to quantify gross changes in CBF haemoglobin parameters that take place between two separate recording sessions. In this instance the change from baseline data generated by the NIRS system was subjected to a second baseline adjustment by creating ‘change from baseline’ data with respect to the 5 min of resting NIRS data collected immediately prior to consumption of the treatment on each day – this provided a more accurate baseline measure of immediately pre-treatment NIRS parameters.

When assessed by NIRS, the increase in cerebral blood flow (CBF) in the surface layers of the cortex during localized neural activity is typically seen as an increase in the concentration of Oxy-Hb and the total concentration of hemoglobin (Total-Hb) and comparative decrease in Deoxy-Hb [40] with both parameters suggested to correspond strongly with the functional magnetic resonance imaging (fMRI) blood oxygen level dependent (BOLD) signal [41, 42].

In this instance, relative changes in the absorption of near-infrared light were measured at a time resolution of 10Hz using a 12-channel Oxymon system (Artinis Medical Systems B.V.). The system emitted two nominal wavelengths of light (~765- and 855 nm) with an emitter/optode separation distance of 4 cm. The differential path-length factor was adjusted according to the age of the participant using the proprietorial software. Relative concentration changes in Oxy-Hb, Deoxy-Hb and Total-Hb were calculated by means of a modified Beer-Lambert law [43] using the proprietorial software. Given the extended recording period and the investigational aims, a simple two emitter/optode pair configuration was utilised (i.e. 2 channels). The emitter/optode pairs were positioned over the left and right frontal cortex using a standard optode holder headband, which separated the pairs from each other by 4 cm. Each pair therefore collected data from an area of prefrontal cortex that included the areas corresponding to the International 10–20 system Fp1 and Fp2 EEG positions. Prior to the primary analysis described below, ANOVAs including hemisphere (i.e. optode) as an additional factor (i.e. hemisphere x day x epoch x treatment) were carried out. In the absence of any treatment related interactions with ‘hemisphere’, data from the two optodes were simply averaged for all of the analyses reported in this paper. The methodology described above been used previously to demonstrate the effects of a number of nutritional interventions on cerebral cerebral blood-flow parameters [44–46].

#### Cognitive tasks

A range of cognitive tasks of varying levels of difficulty, plus a somatically matched control task, were delivered on laptop computers via the Computerised Mental Performance Assessment System (COMPASS) software (Northumbria University, UK). The order of the 5 cognitive tasks and the control task were counterbalanced across participants, with each individual participant completing the tasks in the same order during each assessment. Each task was of 5 min duration, with a two minute resting period between tasks. The selection of tasks comprised:

##### Serial subtractions (3 s, 7 s and 17 s)

Serial subtraction tasks (Serial 3 s and Serial 7 s) have been used previously to elicit hemodynamic responses in the frontal cortex in a number of NIRS [45, 47] and fMRI [48] studies. In this instance modified, five minute versions of the Serial 3 s and Serial 7 s tasks, and a novel Serial 17 s task were employed. For each task a standard instruction screen informed the subject to count backwards in threes (sevens or seventeens) from a given randomly generated number between 800 and 999, as quickly and accurately as possible. Subjects were also instructed verbally that if they made a mistake they should carry on subtracting from the new incorrect number. Each three-digit response was entered via the linear number keys with each digit being represented on screen by an asterisk. Pressing the ‘enter’ key signalled the end of each response and cleared the three asterisks from the screen. The task was scored for total number of subtractions and number of errors. In the case of incorrect responses, subsequent responses were scored as positive if they were correct in relation to the new number.

##### 3-back task

N-Back tasks have been used widely in brain imaging studies probing brain activation, including in the frontal cortex, during working memory tasks [49]. Here the most difficult 3-back version was utilised. A continuous string of letters (upper and lower case; display time 500 msec; inter-stimulus interval of 2.5 s) was presented in the centre of the screen for 5 min. For each stimulus, subjects were instructed to indicate whether this was the same letter that appeared three letters before or not by pressing ‘yes’ and ‘no’ buttons on the response box as rapidly as possible. A third of all stimuli (40/120) represented target pairs. The task was scored for percentage of correct responses and reaction times to correct stimuli.

##### Stroop task

The Stroop task is widely used as a measure of selective attention and executive function. Performance of the task activates multiple brain regions including the frontal cortex [50, 51]. In the five minute computerised version of the Stroop task employed here words describing colours (GREEN, BLUE, RED, YELLOW) were randomly presented in a congruent (e.g., GREEN presented in green text etc.) or incongruent coloured text (e.g., GREEN presented in blue text etc.). For each of the stimuli subjects were instructed to respond to the colour of the text the word was presented in, by pressing one of the four corresponding coloured buttons on the response pad. The task was scored for total % correct and reaction times to correct congruent and incongruent responses.

##### Key tapping control task

A 5 min control task, somatically matched to the task that requires the most physical movement (Serial 3 s) was employed to control for the contribution of physical activity to the measures of metabolism and CBF. Subjects repeatedly made three keyboard key presses at the average rate of performance of the Serial 3 s task, plus 1 standard deviation, for 5 min. The rate of completion was controlled by a metronome tone.

#### Other measures

##### Subjective difficulty and mental fatigue ratings

Visual analogue scales were used to assess the difficulty (“How difficult did you find the task that you have just completed?”) and the subjects’ subjective levels of mental fatigue (“How mentally fatigued do you feel right now?”) following completion of each task. Participants indicated their answer to the question by positioning an ‘x’ on a line anchored “Not at all” and “Extremely” with the mouse/cursor. Each VAS was scored as % along the line towards “Extremely”.

##### Bond-lader mood scales [52]

Prior to and following the cognitive tasks, mood was assessed using ‘Alert’, ‘Content’ and ‘Calm’ factors derived from the Bond-Lader visual analogue mood scales.

##### Energy visual analogue scales (Energy VAS)

Prior to and following the cognitive task period, visual analogue scales were completed which assessed (from ‘very low’ to ‘very high’) the subjects’ self-ratings of ‘concentration’, ‘mental stamina’ and ‘physical stamina’. Scales anchored at either end by ‘not at all’ and ‘extremely’ also assessed the subjects’ self-rated levels of being ‘mentally tired’ and ‘physically tired’. All VAS were scored as % along the line towards ‘very high’/’extremely’.

#### Nutritional status

Nutritional status was assessed before and after the supplementation period with reference to serum/plasma concentrations of homocysteine, vitamin C, vitamin E, vitamin D (25-hydroxy vitamin D_3_), iron, zinc and coenzyme Q10. A total of 18 ml whole blood was collected from each participant in 3 x 6 ml ethylenediaminetetraacetic acid (EDTA). Analysis of the plasma for vitamin E (alpha-tocopherol), coenzyme Q10 (ubichinone-10) and vitamin C (ascorbic acid) was then undertaken using high-performance liquid chromatography (HPLC); homocysteine levels were analysed using chemiluminescence immunoassay (CLIA); and vitamin D (25-hydroxy Vitamin D3) levels were assessed using liquid chromatography-tandem mass spectrometry (LC-MS/MS). A further 4 ml whole blood was collected in 4 ml vacutainer serum tubes for the analysis of iron and zinc levels using photometry. All analyses were undertaken by Labor Dr. Limbach und Kollegen (Heidelberg, Germany).

### Procedure

Participants attended the Brain, Performance and Nutrition Research Centre laboratory (Northumbria University), having fasted for 12 h, on three separate mornings. The practice visit comprised the obtaining of informed consent, the collection of demographic and medical history information and review of conformity to the inclusion and exclusion criteria. Vital signs, height, weight (plus calculation of BMI) were measured, and, where applicable, a confirmatory pregnancy test was carried out. Following screening, participants were instructed in the completion of the cognitive tasks, and were allocated their counterbalanced cognitive task order. They then underwent a full ICa/NIRS/cognitive assessment, as described below for Day 1 and Day 56, with the exception that the ICa and NIRS recording started 10 min before the cognitive task period rather than 70 min before (to allow for treatment absorption) on the subsequent days.

**Fig. 2:**
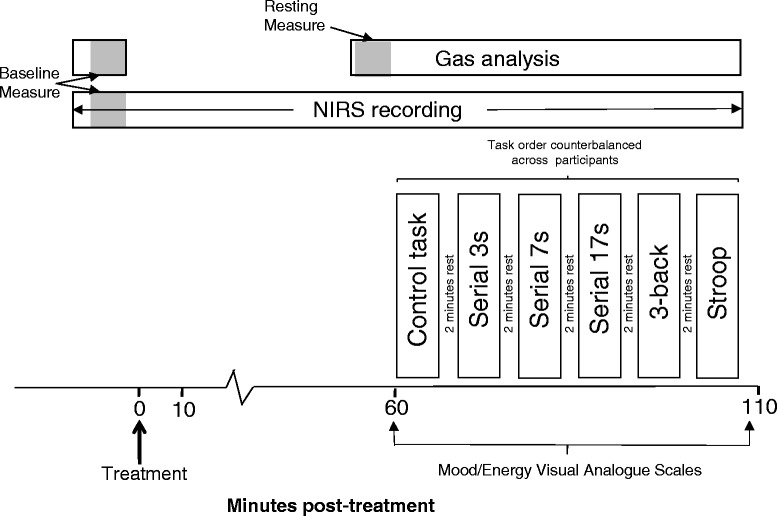
Assessment schedule on Days 1 and 56. Both ICa and NIRS data-collection started with a 10 min pre-dose recording period, the last five minutes of which was used as the baseline measurement. NIRS data was then collected continuously throughout. Following consumption of the day’s treatment participants sat quietly for 60 minutes, with data from the last five minutes of this period used as the ICa resting measurement. Participants then completed the control task, and the five cognitive tasks, in counterbalanced order, with a two minute resting period between each five minute task period

Within 28 days of the practice day participants attended the laboratory for the Day 1 assessment. Following confirmation of compliance with all inclusion/exclusion criteria, and the measurement of vital signs, participants were randomized to their treatment group, after which a venous blood sample was collected. Once the ICa mask and NIRS were in place, and the proper functioning of the equipment had been confirmed, the participant sat for a 10 min resting ICa and NIRS measurement, the last 5 min of which was used as the pre-dose baseline measure for the CBF parameters. Once the participant had consumed their day’s treatment they sat quietly for 60 min, the last 5 min of this period was used to calculate the pre-task, resting-period ICa parameters. Participants then completed the Bond-Lader mood scales and ‘energy’ visual analogue scales. The six x five minute tasks were then completed in the counterbalancing order allocated to the participant, with a two minute rest period between each task. Finally, the participant completed the Bond-Lader mood scales and Energy VAS. NIRS data was collected continuously throughout the entire assessment. The Day 56 assessment was identical to that on Day 1. The running order of the Day 1/56 assessments are shown in Fig. 2.

### Statistical analyses

Prior to commencement the sample size required for the study was calculated using G*Power 3 with respect to the medium effect size (f = 0.24) seen with similar nutritional interventions (e.g. polyphenols) using similar aspects of methodology. In order to exceed power of 0.8 for the measures which were repeated six times per assessment (e.g. ICa) a sample size of 30 participants per group was required, with measures that exceeded this number of repetitions having greater power.

#### Metabolic and CBF parameter data

Data from the metabolic parameters (FatOxi, CarbOxi, TotalEnergy) were collapsed into scores representing the average during 8 separate five minute epochs: comprising, pre-dose baseline, pre-task resting period, control task, and the five cognitive tasks.

The NIRS data, averaged across the left and right optodes, was baseline adjusted with regards the 5 min pre-dose baseline measure for the respective day. Data collected following consumption of the treatment was collapsed (i.e. the mean score was calculated) into: 11 x five minute epochs spanning the 60 min resting absorption period (data collected during completion of the mood scales was discarded), 6 x five minute epochs for the control task and each of the cognitive tasks, and 5 x two minute epochs for each of the rest periods between tasks (see Fig. 4).

Prior to the main analysis. in order to simply confirm that increasing task demands were associated with significant changes in metabolic and NIRS parameters irrespective of treatment, change from resting pre-task baseline data collected on Day 1 and Day 56 were analysed by two-way ANOVA (Day 1/56 x task). These data and results are presented in Additional file 1: Table S1 and Figure S1 in the online materials.

Given the exploratory nature of this study, in order to protect against potential Type II errors, no correction was made for the number of separate linear-mixed models (metabolic data - 3 in total) and ANOVA (NIRS data – 3 in total) analyses described below.

##### Effects of multivitamins/minerals on metabolic parameters

The data for each individual metabolic parameter (FatOxi, CarbOxi, and TotalEnergy) from Day 1 and Day 56 were analysed separately using the linear mixed-effects models (MIXED) procedure in SPSS 21.0. This analysis is appropriate in repeated measures designs that include a baseline covariate as it accounts for the correlation between data points taken from the same subject and allows for missing data across time points (using maximum likelihood to estimate missing data to produce unbiased full datasets with no loss of power). In each of the separate models subject was entered as a random effect and condition, day, task period and their interactions entered as fixed factors. The resting baseline data collected prior to taking the treatment on Day 1 was entered as a covariate in the analysis of each outcome. Post-hoc, Bonferroni adjusted comparisons between the means from each of the multivitamin conditions and the placebo condition were carried out to explore any significant main or interaction effects from the primary analysis.

##### NIRS parameters (Day 1 and Day 56)

The NIRS system utilised here generates ‘change in concentration’ data during a given recording session, rather than quantitative data, and therefore does not generate data that can be meaningfully used as a pre-treatment covariate. The analysis adopted here for each individual NIRS parameter (Oxy-Hb, Deoxy-Hb, Total-Hb) was therefore via separate three-way ANOVAs (Day1/56 x treatment x epoch) of change from baseline (calculated from the relevant day’s immediately pre-treatment rest period) data from each of the 22 epochs spanning the absorption and task periods (see above and Fig. 2.). The primary analysis was via planned comparisons, carried out using t tests calculated with MSError from the ANOVA, which were conducted between placebo and the two multivitamin treatments on means from each epoch. These comparisons were Bonferroni-adjusted to allow for any inflation of Type I errors due to violations of the sphericity assumption [53]. Only those planned comparisons associated with a significant effect on the relevant ANOVA are reported.

A further similar secondary analysis of data solely from the six task periods was conducted to identify any effects of the treatment related to increasing task demands.

#### Cognitive, mental fatigue, task difficulty and mood/energy outcomes

The cognitive outcome data (accuracy and speed of performance from each of five tasks – i.e. 10 outcomes in total) from Day 1 and Day 56 were explored with individual two-way (treatment x day) ANOVAs. Subjective mental fatigue and difficulty ratings acquired after performance of the tasks were explored with two-way (treatment x task) mixed ANOVAs of data from the individual day. Mood/energy outcome data were analysed with two-way (treatment x before/after tasks) mixed ANOVAs of data from the individual days. Post-hoc comparisons (Bonferroni adjusted) were conducted on data from each day for those measures that reached significance.

#### Nutritional parameters

Analysis of the nutritional blood analyte parameters was primarily by one-way ANCOVA of Day 56 data, using the corresponding Day 1 (pre-treatment) data as covariate. Separate one-way ANOVAs of Day 1 and Day 56 data were also conducted to confirm the equivalence of the groups pre-treatment, and post-treatment group differences on any measure.

## Results

Data and results with regards the effects of differing task demands on all subjective, metabolic and CBF outcomes, irrespective of treatment, are presented in the online materials in Additional file 1: Figure S1 and Table S1.

### Effects of multivitamins/minerals on metabolic parameters

*Fat oxidation –* there was a significant interaction between treatment and Day 1/56 [F (2, 270) = 6.393, *P* < 0.05]. Reference to the post-hoc comparisons between means showed that Fatoxi was significantly increased following a single dose of the 3RDA treatment on Day 1 (*p* < 0.01), with no significant differences evident after 8 weeks administration (Day 56).

*Carbohydrate oxidation* – there was a significant interaction between treatment and Day 1/56 [F (2, 276) = 9.9, *P* < 0.01]. However, this effect represented opposite, non-significant patterns on the two days, and reference to the post-hoc comparisons showed that there were no significant differences between the active treatment and placebo means on either day (all, P > 0.1).

*Total energy* - there was a significant main effect of treatment with regards Total Energy [F (2, 82 = 4.354, *P* < 0.05] with consumption of the 3RDA treatment resulting in increased energy expenditure during task performance both after a single dose and 8 weeks supplementation [Day 1 (*P* < 0.05), Day 56 (*P* < 0.01)].

**Fig. 3:**
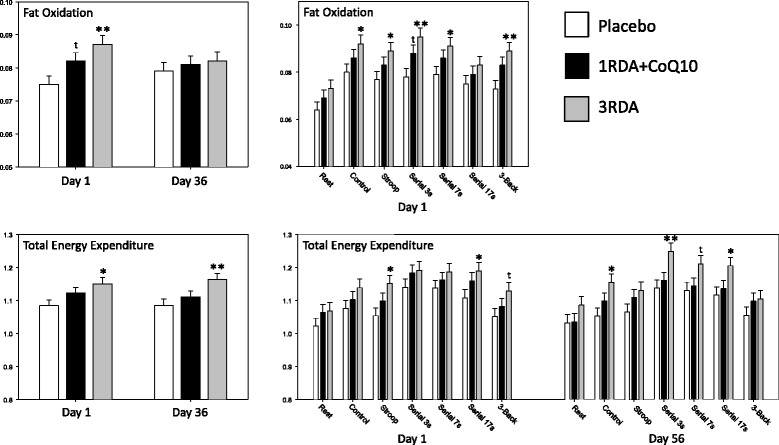
Acute and chronic effects of the multivitamin treatments on metabolism. with Bonferroni corrected post-hoc comparisons to placebo. The graphs on the left show the treatment x day interaction with regards FatOxi and the main effect of treatment on TotalEnergy. The graphs on the right show the data broken down by task for illustrative purposes. All data are adjusted means (+SEM), derived from the linear mixed-effects models analysis incorporating the Day 1, pre-treatment baseline measure as a covariate. t, *p* <0.1; *, *p* < 0.05; **, *p* < 0.01; ***, *p* < 0.001 from the post-hoc comparisons between the two active treatments and placebo

Significant effects on the ICa measures are presented graphically in Fig. 3, with an additional illustrative graphic representation of the data for each task period. Data are presented in online Additional file 1: Table S2.

### Effects of multivitamins/minerals on CBF parameters

Reference to the ANOVA of the combined Day 1/Day 56 NIRS data showed that there was a significant interaction between epoch, day and treatment for Total-Hb [F (42,1617) = 1.521, *P* < 0.05]. Reference to the Bonferroni adjusted planned comparisons of data from each of the 22 epochs showed that CBF, as indexed by Total-Hb concentrations, was significantly increased during the 2^nd^, 3^rd^ , 5^th^ and 6^th^ task periods (all *P* < 0.01) on the 1^st^ day of treatment following the 1RDA + Q10 treatment. There were no significant differences associated with the 3RDA treatment or during any epoch following either treatment on Day 56.

The pattern of effects in terms of Oxy-Hb was largely the same (epoch x day x treatment interaction, [F(42,1617) = 1.396, *P* < 0.05]). Following a single dose of the 1RDA + Q10 treatment, concentrations of Oxy-Hb were increased during the 2^nd^, 3^rd^, 5^th^ and 6^th^ task periods (all *P* < 0.01) on Day 1. Additionally Oxy-Hb was also elevated during the rest period between the 5^th^ and 6^th^ task periods following 1RDA + Q10 (*P* < 0.05) and during a single task period following the 3RDA treatment (1st Day, 6^th^ task period - *P* < 0.01). There were no significant differences during any epoch following either treatment on Day 56.

**Fig. 4:**
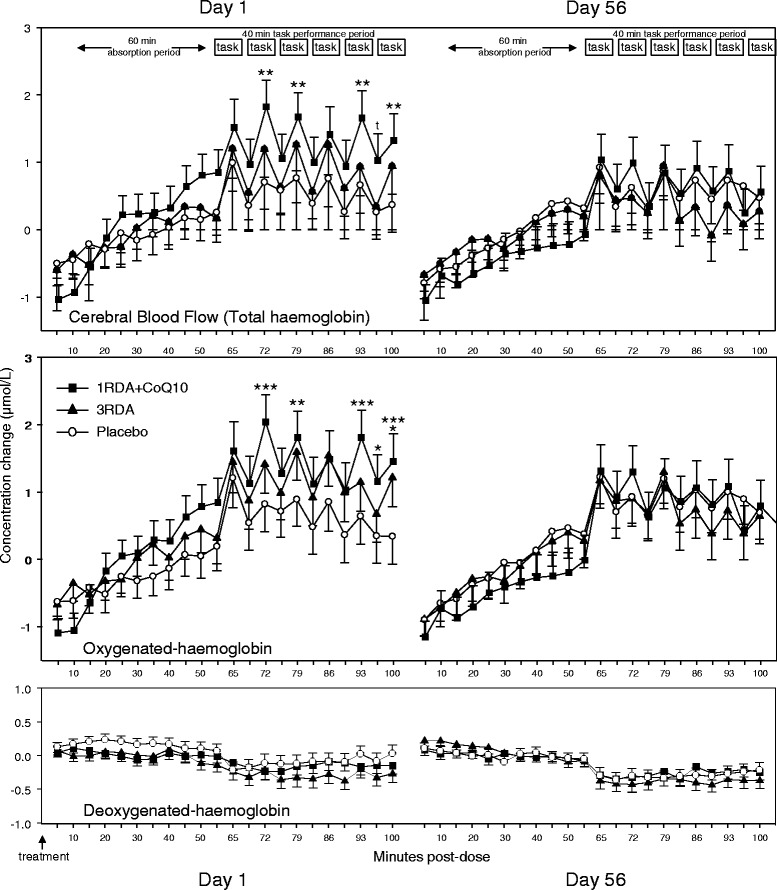
Mean (+SEM) Total-Hb, Oxy-Hb and Deoxy-Hb data from each NIRS recording epoch on Day 1 and Day 56 baseline adjusted to a pre-treatment resting period. Following a 60 minute absorption period participants completed a control key-tapping task and 5 cognitive tasks (all 5 min duration) in counterbalanced order with a 2 min resting period between each task. t, *p* < 0.1; *, *p* < 0.05; **, *p* < 0.01; ***, *p* < 0.001, from Bonferroni adjusted comparisons between placebo and the two multivitamin treatments during each epoch

There were no significant effects either with regards Deoxy-Hb. Mean data (+ SEM) for each epoch on both days for Total-Hb, Oxy-Hb and Deoxy-Hb are shown in Fig. 4.

### Effects of multivitamins/minerals on cognitive performance, mood and VAS

There were no significant differences in task performance following either acute or chronic administration of the treatments with the exception of a main effect on 3-Back Correct across both days [F (2,81) = 4.21, *P* < 0.05]. Reference to the comparisons between placebo and treatment means showed that this effect represented more correct responses following placebo in comparison to the 1RDA + Q10 treatment (both days, *P* < 0.05). The cognitive data are presented in Additional file 1: Table S3 in the online materials.

There were no significant effects of treatment on ratings of either mental fatigue or task difficulty following a single dose (Day 1) or mental fatigue following 8 weeks administration. However, there was a significant difference evinced in terms of perceived difficulty ratings on Day 56 (treatment x task interaction, [F (10,365) = 2.31, *P* < 0.05]), with both multivitamin treatment groups (*P* < 0.01) rating the 3-Back task as having been more difficult in comparison to the placebo group. However, it may be notable that this finding was as a result of a sharp drop in difficulty ratings (as compared to other tasks and data from Day 1) restricted to this one task in the placebo condition. Subjective difficulty and mental fatigue outcome data (+SEM) for Day 1 and Day 56 are presented in Additional file 1: Table S4 in the online materials.

There were no significant effects on the Bond-Lader mood scales or Energy VAS.

#### Nutritional status parameters

ANOVA of baseline data (Day 1 pre-dose) showed that the groups did not differ significantly in terms of any nutritional status parameter at the outset of the study. However, there was a trend towards such an effect with regards CoQ10 levels, with inspection of the groups means suggesting that this was as a consequence of numerically higher levels in the 1RDA + Q10 group than the other two groups prior to treatment. The primary analysis (ANCOVA of Day 56 data using Day 1 as covariate) showed that by Day 56 both vitamin D3 and homocysteine levels were significantly modulated by treatment (vitamin D3 [F (2,75) = 4.13, *P* < 0.05], homocysteine [F (2,75) = 8.3, *p* < 0.001]). Both multivitamin treatments resulted in elevated vitamin D3 levels (1RDA + CoQ10, *P* < 0.05 – 3RDA, *P* < 0.01]) and reduced homocysteine levels (1RDA + CoQ10, *P* < 0.05 – 3RDA, *P* < 0.001]). A confirmatory analysis of micronutrient levels using data solely from Day 56 showed that, as well as significantly higher levels of vitamin D3 and lower levels of homocysteine in both multivitamin groups, CoQ10 levels were also significantly higher in the 1RDA + Q10 group post-treatment (*P* < 0.05). Data for the nutritional parameters from Day 1 and Day 56 (+SEM) are shown in Table 3.

**Table 3:** Mean nutritional parameter data (+SEM) from Day 1 and Day 56

			Day 1		Day 56		Day 56 ANCOVA	
	Treatment	N	Mean	SEM	Mean	SEM	Adjusted mean	SEM
Vitamin C (mg/L)	1RDA + Q10	24	5.30	0.51	6.02	1.23	6.23	0.62
	3RDA	26	6.87	1.04	6.42	1.26	6.30	0.59
	Placebo	29	6.62	0.61	5.21	0.97	5.14	0.56
Vitamin D (mg/L)	1RDA + Q10	24	25.41	2.40	30.5^a^	6.22	28.73*	1.51
	3RDA	26	24.27	2.19	30.1^a^	5.90	29.23*	1.45
	Placebo	29	20.22	2.22	21.76	4.04	23.97	1.39
Vitamin E (mg/L)	1RDA + Q10	24	11.61	0.56	12.1	2.47	12.00	0.40
	3RDA	26	11.73	0.55	12.55	2.46	12.36	0.39
	Placebo	29	11.12	0.44	11.36	2.11	11.62	0.37
CoQ10 (μg/L)	1RDA + Q10	24	562.3	46.0	586.3^a^	119.7	541.8	36.64
	3RDA	26	473.1	33.3	452.5	88.8	463.4	34.41
	Placebo	29	447.1	26.3	437.8	81.3	464.8	32.89
Iron (μmol/L)	1RDA + Q10	24	1010.7	75.8	951.0	194.1	954.2	75.41
	3RDA	26	1064.4	100.1	1054.9	206.9	1042.4	72.55
	Placebo	29	992.9	89.6	985.2	183.0	993.7	68.65
Zinc (μmol/L)	1RDA + Q10	24	13.16	0.52	13.65	2.79	13.66	0.31
	3RDA	26	13.13	0.28	13.34	2.62	13.35	0.30
	Placebo	29	13.26	0.22	13.23	2.46	13.22	0.28
Homocysteine (μmol/L)	1RDA + Q10	24	11.79	0.61	10.54	2.15	10.35*	0.45
	3RDA	26	11.83	0.75	9.5^a^	1.86	9.30*	0.43
	Placebo	29	10.76	0.41	11.36	2.11	11.71	0.41

Footnote: a = *p* < 0.05 from t test comparing means for measures that evinced a significant difference on one way ANOVA of data from the individual day. * = *p* < 0.05 from t test comparing means for measures that evinced a significant difference on one way ANCOVA of data from Day 56 using Day 1 data as covariate

## Discussion

The results of the current study demonstrate that the administration of single doses of vitamins and minerals (± CoQ10) can modulate whole-body metabolic parameters and frontal cortex CBF hemodynamic responses during the performance of cognitive tasks of graded difficulty. The modulation of metabolic parameters was also seen following 8 weeks administration.

With regards to the metabolic parameters, the multivitamin/mineral treatment had a dose dependent effect, with significant modulation only achieved following the treatment containing the higher (3RDA) of two doses of water soluble vitamins. This treatment resulted in both increased fat oxidation (FatOxi) and increased total energy expenditure (TotalEnergy), as assessed using indirect calorimetry of exhaled pulmonary gas. These effects were seen throughout the cognitive task period which commenced 60 min post-dose. The increase in total energy expenditure was also sustained, and consolidated, at the end of the 8 week treatment period. Whilst not having a significant impact on metabolism, the lower dose of vitamins/minerals with added coenzyme Q10 (1RDA + Q10) increased the CBF hemodynamic response in the frontal cortex during tasks that activate this brain area following a single dose. In this case both Total-Hb, a measure of overall local CBF, and Oxy-Hb were increased, with no significant effect on concentrations of Deoxy-Hb. Interestingly, the pattern of hemodynamic responses was similar, if less striking, in the 3RDA group (and reached statistical significance during several tasks prior to the Bonferroni corrections). There was no evidence of acute modulation of hemodynamic parameters as a consequence of the day’s dose of the treatment after 8 weeks administration of either treatment. However, as noted above, CW-NIRS generates concentration change, rather than quantitative data, and therefore only provides a measure of acute changes in CBF hemodynamics during each discrete recording session. It therefore provides no direct measure of any changes that have taken place between recording sessions, in this case as a consequence of chronic supplementation with micronutrients. The lack of a chronic effect may therefore reflect a simple attenuation of the acute effects seen following the first dose of 1RDA + Q10 on Day 1. However, this could equally, in turn, reflect either increased circulating levels of micronutrients as a consequence of 8 weeks administration, which may have precluded a further acute effect of an additional dose of micronutrients on Day 56; or it may indicate that a gross, undetectable change in CBF parameters had already taken place, attenuating the possibility of any additional acute effects of Day 56’s treatment.

Whilst this study also provides the first demonstration that simply increasing the demands of cognitive tasks significantly modulates whole-body metabolic parameters, there was no significant interaction between treatment and specific tasks for either the metabolic or CBF parameters.

In general, the results with regards the effects of micronutrients on energy metabolism are broadly in line with those of Li et al. [16], who examined the effect of 26 weeks supplementation with multivitamin/minerals on overall resting metabolism in obese females using ICa, and found that micronutrient supplementation was associated with a significant increase in resting energy expenditure and fat oxidation. The specific acute, dose-related increase in fat oxidation seen here is also consistent with previous ICa studies that have demonstrated an increase in fat oxidation as a consequence of acute and chronic administration of calcium [54], acute supplementation with calcium and vitamin D [55] and chronic vitamin C supplementation [56]. In terms of the mechanisms underlying the effects on overall energy expenditure it is notable that vitamins as a group are directly involved in every aspect of mitochondrial function including aerobic respiration and cellular energy production [10–14]. With regards fat oxidation, the effects of calcium have been attributed in part to modulation of lipolysis and lipogenesis driven by modulation of levels of circulating 1,25-(OH)_2_D_3_ and parathyroid hormone [57], whereas vitamin C is theorised to have its effect via its role as a cofactor for the biosynthesis of carnitine, an essential molecule in the oxidation of fatty acids [56]. Whilst the effects on fat metabolism here may be predicated simply on the inclusion of calcium, and vitamins C and D in the micronutrient preparations, it is not possible to preclude modulation of a multitude of metabolic parameters by the other vitamin and mineral components. It is also worth noting that whereas the chronic effects of supplementation on fat oxidation were not significant, the numeric pattern of the data was consistent with the acute results.

The pattern of CBF parameter effects seen here was for a treatment related augmentation of the hemodynamic responses seen during the performance of the active cognitive tasks, with significant increases in total-haemoglobin, a proxy measure of CBF, seen during four of the five active task periods. In this instance, due to the counterbalancing of the order of task performance, the benefits could not be attributed to specific task demands, and a secondary task x treatment analysis did not demonstrate an interaction between the two factors. The modulation of CBF parameters seen here may reflect the requirement to deliver more metabolic substrates to active neural tissue to support the increased energy metabolism during task performance, although the lack of the linear dose–response that was seen with regards the fat/energy metabolism measures (for which the 3RDA elicited greater modulation) with respect to the NIRS measures argues against this. Rather, the increased hemodynamic response may well be driven by direct modulation of the neurovascular coupling of local blood flow to activity. In line with this, the cardiovascular/vasodilatory properties of several micronutrient components of the interventions here, including vitamin C [24, 25], vitamin B_9_ [28] and CoQ10 [32], have previously been attributed to their roles in the synthesis of nitric oxide, the ubiquitous signalling molecule that mediates both peripheral vasodilation and neurovascular coupling [3, 4]. The results here certainly find support from those previous studies that demonstrated improved peripheral vasodilation (as assessed by flow mediated dilatation), following single doses of vitamins B_9_, C and E [20–23]. Naturally, the question arises as to why the CBF effects were greater in the treatment containing lower levels of water soluble vitamins. The obvious answer is that the 1RDA treatment also contained CoQ10, which is intricately intertwined with the working of the vitamins with respect to mitochondrial function [15], and which may also exert an independent effect in terms of NO synthesis [32]. Supplementation with CoQ10 has been shown to consistently engender improvements in peripheral endothelial function and other cardiovascular parameters [32], which would be expected to co-vary with modified brain vasodilation. Having said this, the dose administered here was comparatively small; 4.5 mg as opposed to 150 mg + in most of the previous cardiovascular studies. This suggests that the doses administered in the cardiovascular studies are unnecessarily large, or that co-administration with vitamins relevant to CoQ10 function engenders a synergistic effect. Either way, reference to the nutritional parameter data showed that the 1RDA + Q10 group had significantly greater plasma levels of CoQ10 than the other treatment groups after 56 days supplementation, suggesting that this dose was adequate to augment the levels of CoQ10 that were being synthesised endogenously by individuals during the supplementation period.

It is also noteworthy that single doses of multi-vitamin/minerals have previously been shown to modulate regional brain activity during a task measuring focussed attention as measured with functional magnetic resonance spectroscopy (fMRI) [58], and cerebro-electrical activity during an attention task as measured by electroencephalography (EEG) [59].

It is also worth noting that, in this instance, visual inspection of the data in Fig. 4 shows that the pattern of concentration change in haemoglobin concentrations was for a gradual increase during the absorption period following all treatments. However, this pattern represents concentrations simply returning to approximately zero over this period i.e. reverting to the resting baseline values. We surmise that the initial reductions in haemoglobin concentrations immediately after the treatments reflect the break in ‘quiet’ sitting and the consumption of the treatment capsules plus water at this point. Given that all of the reported significant effects of treatment are directly in comparison to placebo, and that these are seen only during task completion, it seems unlikely that any underlying pattern of gradual modulation during the absorption period could have influenced the results reported here.

In terms of cognitive function, there was no interpretable evidence of modulated performance. Whilst a single measure evinced an isolated statistical difference, there was no evidence of similar effects from any other measure, so it seems likely this was just a chance finding. The lack of cognitive effects may be attributable to several factors; this was a comparatively small study with less than 30 participants per group (as opposed to over 100 per group in previous cognitive studies showing benefits of multivitamins/minerals [37, 38]; the participants were fitted with restrictive head gear and a face mask, potentially introducing physical or psychological noise into the data; and the cognitive outcomes did not have a baseline that could be used as a covariate, greatly reducing the sensitivity of the assessment.

With regards the nutritional parameters, Vitamin D was raised following 56 days supplementation with both multivitamin interventions, and plasma levels of CoQ10 were significantly higher in the 1RDA + Q10 group on Day 56 (although this did not represent a significant increase on the ANCOVA, possibly due to a trend towards higher pre-treatment levels in this group on Day 1). Homocysteine levels were also reduced by both treatments. This is of particular interest as this potentially neurotoxic amino acid, levels of which correlate positively with cardiovascular disease, age-associated cognitive decline and dementia [34], can be seen as a marker of low endogenous levels of a number of B vitamins. The mean pre-treatment homocysteine levels in this study were ~11.5 μmol/L on Day 1, a figure that is close to the 11.7 μmol/L reported as the average in the UK’s Low Income Diet and Nutrition Survey [57]. The cut-off point in homocysteine levels associated with greater cardiovascular disease risk is typically taken as 12 μmol/L. Some 35 % of the current study’s sample exceeded this cut-off. These figures, and the fact that supplementation with simple multi-vitamins significantly reduced homocysteine levels in a dose-related manner (to 10.5 and 9.5 μmol/L respectively for the 1RDA + Q10 and 3RDA treatments), suggest strongly that the cohort here, who were not selected on the basis of any nutritional parameters, did not have optimal, or even adequate, nutritional status as a group at the study outset. Similarly, pre-treatment vitamin D levels in the cohort of ~20–25 mg/L would be regarded as insufficient by some established cut-off parameters, suggesting that a dose of 200 IU is adequate to rectify this insufficiency.

With regards the limitations of the study, the most obvious is that one of the working hypotheses here was that the treatments might have a greater effect on CBF parameters and metabolism during the more difficult tasks. The individual tasks impacted on CBF parameters and metabolism depending on their cognitive demands, but the effects of the micronutrients were seen as main effects or interaction effects of treatment, with no task interactions. In hindsight a more comprehensive assessment of simple resting metabolism would have been informative. Having said this, the study provides a very useful starting point for future studies looking at the metabolic effects of micronutrients, and in particular it demonstrates that ICa is a sensitive measure of the somatic metabolic effects of brain activity. A second limitation of the study was the inability of the NIRS equipment utilised here to measure quantitative changes over time. This makes the absence of acute effects on Day 56 difficult to interpret, but again, suggests that research using the more recently introduced quantitative NIRS systems, which can be used to measure changes over time, would be informative.

## Conclusions

In summary, the results of the current study suggest that simple supplementation with micronutrients, many of which are implicated in fat oxidation, mitochondrial metabolism and vasodilation, can increase fat oxidation, total somatic energy expenditure and cerebral blood-flow during task performance following a single dose, and increase energy expenditure following 8 weeks supplementation. These results suggest several conclusions. The first is that single doses of multi-vitamins/minerals are capable of modulating metabolic parameters, and that, furthermore, a single dose of multi-vitamins/minerals with added CoQ10 can increase the CBF response to task performance in the frontal cortex, thereby increasing the delivery of metabolic substrates to active neural tissue. Given that the higher 3RDA dose of water soluble vitamins failed to elicit significant modulation of oxygenated and total-haemoglobin these results suggest that the dosage of CoQ10 (4.5 mg) administered here was sufficient to act either additively or synergistically with the co-administered vitamins/minerals in terms of promoting cerebral vasodilation. Second, the results suggest that multivitamin/mineral supplementation over a longer period of time (in this case 56 days) is capable of significantly increasing overall energy metabolism/expenditure. Given that there was no interaction between task demands and treatment related effects, it is possible that these effects could also be seen at rest, and likely that they would be evident during physical exercise. These possibilities deserve further research attention.

Finally, the logical conclusion that can be garnered from the results here is that, if it is possible to beneficially modulate core physiological processes such as energy metabolism and cerebral blood-flow by simply administering vitamins and other micronutrients to healthy members of the population, then it must be the case that the nutritional status of the sample, and by implication the general population, is inadequate. This suggestion is supported by the comparatively high pre-treatment levels of homocysteine seen across the cohort here. If it is the case that our results reflect wide-spread nutritional insufficiency, and given the challenge that shifting the general population’s dietary patterns poses, then, in the absence of a healthy diet, supplementation with vitamins and other micronutrients may prove a useful method of recharging nutritional status for a section of the population.

## Additional file

Additional file 1: Figure S1. Exploratory analysis of the effects of differing task demands on ratings of mental fatigue/difficulty and all physiological variables, irrespective of treatment. **Table S1.** Mean scores (+SEM) averaged across Day 1 and Day 56 from the exploratory analysis of the effects of differing task demands on ratings of mental fatigue/difficulty and all physiological variables, irrespective of treatment. **Table S2.** Means (+SEM) of ICa data obtained during the 5 min pre-task resting period, the control tapping task and 5 task periods on Day 1 and Day 56. **Table S3.** Cognitive task performance scores (mean plus SEM) on Day 1 and Day 56. **Table S4.** Subjective difficulty and mental fatigue data (+SEM) from Day 1 and Day 56. (DOC 328 kb)

## Abbreviations

BMI: body mass index
BOLD: blood oxygen level dependent
CarbOxi: carbohydrate oxidation
CBF: cerebral blood-flow
CLIA: chemiluminescence immunoassay
CoQ10: coenzyme Q10
Deoxy-Hb: deoxygenated hemoglobin
EDTA: ethylenediaminetetraacetic acid
FatOxi: fat oxidation
HPLC: high-performance liquid chromatography
ICa: indirect calorimetry
LC-MS/MS: liquid chromatography-tandem mass spectrometry
NIRS: near-infrared spectroscopy
Oxy-Hb: oxygenated hemoglobin
RDA: recommended dietary allowances
TotalEnergy: total energy expenditure
Total-Hb: total hemoglobin
VAS: visual analogue scales
