# Supplementary material for: Multivitamins and minerals modulate whole-body energy metabolism and cerebral blood-flow during cognitive task performance: a double-blind, randomised, placebo-controlled trial
Source: Nutr Metab (Lond). 2016 Feb 11;13:11. doi: 10.1186/s12986-016-0071-4 (PMC4750202; doi:10.1186/s12986-016-0071-4)
Supplement: Additional file 1: Figure S1. — Exploratory analysis of the effects of differing task demands on ratings of mental fatigue/difficulty and all physiological variables, irrespective of treatment. Table S1. Mean scores (+SEM) averaged across Day 1 and Day 56 from the exploratory analysis of the effects of differing task demands on ratings of mental fatigue/difficulty and all physiological variables, irrespective of treatment. Table S2. Means (+SEM) of ICa data obtained during the 5 min pre-task resting period, the control tapping task and 5 task periods on Day 1 and Day 56. Table S3. Cognitive task performance scores (mean plus SEM) on Day 1 and Day 56. Table S4. Subjective difficulty and mental fatigue data (+SEM) from Day 1 and Day 56. (DOC 328 kb) [file 12986_2016_71_MOESM1_ESM.doc]

**Online Supplemental Material**

Supplemental Figure 1. Exploratory analysis of the effects of differing task demands on ratings of mental fatigue/difficulty and all physiological variables, irrespective of treatment. Analysis was by two-way (task x day) ANOVA of either raw scores (mental fatigue/difficulty) or change from baseline data (adjusted to a 5 min pre-task resting measurement) from both Day 1 and Day 56. Data are means during each task period (+SEM) and are organized by increasing ‘subjective difficulty’ (bottom right). *, p < 0.05; **, p < 0.01; ***, p < 0.001 from Bonferroni adjusted post-hoc comparisons of each task to the somatically matched (key tapping) control task. Main effects of ‘task’ from the ANOVAs are presented in each graph.

|  | **Subjective difficulty**  (%VAS) | | | | **Mental fatigue**  (%VAS) | | | | **Heart rate**  (bpm) | | | |
| --- | --- | --- | --- | --- | --- | --- | --- | --- | --- | --- | --- | --- |
|  | Mean | SEM | T(335) | P | Mean | SEM | T(335) | P | Mean | SEM | T(190) | p |
| Control task | 23.95 | 1.46 |  |  | 46.10 | 1.879 |  |  | -0.524 | 1.155 |  |  |
| Stroop task | 32.92 | 1.73 | 3.58 | < 0.01 | 47.91 | 1.868 | 0.88 | ns | 0.162 | 1.146 | 1.57 | ns |
| Serial 3s | 40.53 | 1.71 | 6.61 | < 0.001 | 51.64 | 1.791 | 2.69 | < 0.05 | 1.206 | 1.169 | 3.96 | < 0.01 |
| Serial 7s | 52.15 | 1.67 | 11.24 | < 0.001 | 54.36 | 1.726 | 4.02 | < 0.001 | 1.627 | 1.191 | 4.93 | < 0.01 |
| Serial 17s | 61.08 | 1.61 | 14.80 | < 0.001 | 53.09 | 1.861 | 3.40 | < 0.01 | 1.834 | 1.188 | 5.40 | < 0.01 |
| 3-Back | 62.08 | 1.81 | 15.20 | < 0.001 | 55.08 | 1.850 | 4.36 | < 0.001 | 0.554 | 1.116 | 2.47 | ns |
|  |  | | | |  | | | |  | | | |
|  | **Fat oxidation**  **(g/min)** | | | | **Carbohydrate oxidation**  **(g/min)** | | | | **Total energy expenditure (kcal/min)** | | | |
|  | Mean | SEM | T(395) | p | Mean | SEM | T(360) | p | Mean | SEM | T(360) | p |
| Control task | 0.027 | 0.002 |  |  | -0.044 | 0.004 |  |  | 0.060 | 0.010 |  |  |
| Stroop task | 0.022 | 0.002 | 2.34 | ns | -0.035 | 0.004 | 1.90 | ns | 0.060 | 0.012 | 0.05 | ns |
| Serial 3s | 0.028 | 0.002 | 0.17 | ns | -0.026 | 0.005 | 3.85 | < 0.001 | 0.135 | 0.013 | 4.60 | < 0.001 |
| Serial 7s | 0.026 | 0.002 | 0.74 | ns | -0.025 | 0.004 | 3.97 | < 0.001 | 0.121 | 0.014 | 3.71 | < 0.01 |
| Serial 17s | 0.019 | 0.002 | 3.52 | < 0.01 | -0.015 | 0.005 | 6.25 | < 0.001 | 0.112 | 0.014 | 3.20 | < 0.01 |
| 3-Back | 0.020 | 0.002 | 3.19 | < 0.01 | -0.032 | 0.004 | 2.49 | ns | 0.042 | 0.011 | 1.16 | ns |
|  |  | | | |  | | | |  | | | |
|  | **Oxygenated haemoglobin**  (Δµmol/L) | | | | **Deoxygenated haemoglobin**  (Δµmol/L) | | | | **Total haemoglobin**  (Δµmol/L) | | | |
|  | Mean | SEM | T(395) | p | Mean | SEM | T(395) | p | Mean | SEM | T(395) | p |
| Control task | 0.569 | 0.190 |  |  | -0.095 | 0.060 |  |  | 0.474 | 0.185 |  |  |
| Stroop task | 0.511 | 0.186 | 0.32 | ns | -0.140 | 0.060 | 0.75 | ns | 0.371 | 0.190 | 1.73 | ns |
| Serial 3s | 1.554 | 0.194 | 5.36 | < 0.001 | -0.219 | 0.058 | 2.07 | ns | 1.335 | 0.188 | 14.48 | < 0.001 |
| Serial 7s | 1.627 | 0.191 | 5.76 | < 0.001 | -0.297 | 0.061 | 3.38 | < 0.01 | 1.330 | 0.190 | 14.39 | < 0.001 |
| Serial 17s | 1.738 | 0.198 | 6.37 | < 0.001 | -0.346 | 0.062 | 4.22 | < 0.001 | 1.393 | 0.189 | 15.44 | < 0.001 |
| 3-Back | 0.927 | 0.199 | 1.95 | ns | -0.312 | 0.057 | 3.63 | < 0.01 | 0.615 | 0.193 | 2.38 | ns |

Supplemental Table 1. Mean scores (+SEM) averaged across Day 1 and Day 56 from the exploratory analysis of the effects of differing task demands on ratings of mental fatigue/difficulty and all physiological variables, irrespective of treatment. Data for ratings of mental fatigue/difficulty are raw scores and all other variables are change from baseline acores (adjusted to a 5 min pre-task resting measurement). Also presented are T scores (df) and probabilities with respect to Bonferroni adjusted comparisons between the control task and each other task.

Supplemental Table 2. Means (+SEM) of ICa data obtained during the 5 min pre-task resting period, the control tapping task and 5 task periods on Day 1 and Day 56. Data are adjusted means derived from the linear mixed-effects models analysis incorporating the Day 1 pre-treatment baseline measure as covariate for each task period. The total number of participants providing data for the analysis were, placebo – 30, 1RDA+Q10 – 29, 3RDA – 26.

|  |  | Day 1 | | | | | | Day 56 | | | | | |
| --- | --- | --- | --- | --- | --- | --- | --- | --- | --- | --- | --- | --- | --- |
|  |  | Carbohydrate  oxidation  (g/min) | | Fat  oxidation  (g/min) | | Total energy  expenditure  (Kcal/min) | | Carbohydrate  oxidation  (g/min) | | Fat  oxidation  (g/min) | | Total energy  expenditure  (Kcal/min) | |
| Task period | Treatment | Mean | SEM | Mean | SEM | Mean | SEM | Mean | SEM | Mean | SEM | Mean | SEM |
| Resting | Placebo | 0.111 | 0.0074 | 0.064 | 0.0034 | 1.022 | 0.024 | 0.103 | 0.0078 | 0.069 | 0.0036 | 1.032 | 0.0252 |
|  | 1RDA+Q10 | 0.111 | 0.0076 | 0.069 | 0.0035 | 1.063 | 0.024 | 0.109 | 0.0078 | 0.068 | 0.0036 | 1.035 | 0.0248 |
|  | 3RDA | 0.103 | 0.0080 | 0.073 | 0.0037 | 1.068 | 0.025 | 0.116 | 0.0081 | 0.069 | 0.0038 | 1.086 | 0.0261 |
| Control task | Placebo | 0.086 | 0.0078 | 0.08 | 0.0036 | 1.075 | 0.025 | 0.075 | 0.0074 | 0.084 | 0.0034 | 1.053 | 0.0238 |
|  | 1RDA+Q10 | 0.084 | 0.0078 | 0.086 | 0.0036 | 1.102 | 0.025 | 0.081 | 0.0076 | 0.087 | 0.0035 | 1.098 | 0.0241 |
|  | 3RDA | 0.078 | 0.0081 | 0.092 | 0.0038 | 1.138 | 0.026 | 0.09 | 0.0080 | 0.089 | 0.0037 | 1.154 | 0.0255 |
| Stroop | Placebo | 0.09 | 0.0074 | 0.077 | 0.0034 | 1.055 | 0.024 | 0.087 | 0.0078 | 0.08 | 0.0036 | 1.066 | 0.0252 |
|  | 1RDA+Q10 | 0.091 | 0.0076 | 0.083 | 0.0035 | 1.1 | 0.024 | 0.095 | 0.0077 | 0.081 | 0.0035 | 1.11 | 0.0246 |
|  | 3RDA | 0.089 | 0.0080 | 0.089 | 0.0037 | 1.152 | 0.025 | 0.096 | 0.0081 | 0.083 | 0.0038 | 1.132 | 0.0261 |
| Serial 3s | Placebo | 0.097 | 0.0074 | 0.073 | 0.0034 | 1.052 | 0.024 | 0.094 | 0.0078 | 0.075 | 0.0036 | 1.056 | 0.0252 |
|  | 1RDA+Q10 | 0.085 | 0.0076 | 0.083 | 0.0035 | 1.082 | 0.024 | 0.094 | 0.0078 | 0.08 | 0.0036 | 1.099 | 0.0248 |
|  | 3RDA | 0.084 | 0.0080 | 0.089 | 0.0037 | 1.129 | 0.025 | 0.103 | 0.0081 | 0.077 | 0.0038 | 1.105 | 0.0260 |
| Serial 7s | Placebo | 0.105 | 0.0074 | 0.079 | 0.0034 | 1.137 | 0.024 | 0.096 | 0.0078 | 0.083 | 0.0036 | 1.13 | 0.0252 |
|  | 1RDA+Q10 | 0.099 | 0.0076 | 0.086 | 0.0035 | 1.161 | 0.024 | 0.099 | 0.0078 | 0.085 | 0.0036 | 1.143 | 0.0248 |
|  | 3RDA | 0.094 | 0.0080 | 0.091 | 0.0037 | 1.186 | 0.025 | 0.112 | 0.0081 | 0.085 | 0.0037 | 1.21 | 0.0258 |
| Serial 17s | Placebo | 0.108 | 0.0078 | 0.075 | 0.0036 | 1.107 | 0.025 | 0.099 | 0.0074 | 0.08 | 0.0034 | 1.117 | 0.0238 |
|  | 1RDA+Q10 | 0.114 | 0.0078 | 0.079 | 0.0036 | 1.159 | 0.025 | 0.108 | 0.0076 | 0.079 | 0.0035 | 1.136 | 0.0241 |
|  | 3RDA | 0.111 | 0.0081 | 0.083 | 0.0037 | 1.189 | 0.026 | 0.132 | 0.0080 | 0.076 | 0.0037 | 1.205 | 0.0255 |
| 3-back | Placebo | 0.097 | 0.0074 | 0.073 | 0.0034 | 1.052 | 0.024 | 0.094 | 0.0078 | 0.075 | 0.0036 | 1.056 | 0.0252 |
|  | 1RDA+Q10 | 0.085 | 0.0076 | 0.083 | 0.0035 | 1.082 | 0.024 | 0.094 | 0.0078 | 0.08 | 0.0036 | 1.099 | 0.0248 |
|  | 3RDA | 0.084 | 0.0080 | 0.089 | 0.0037 | 1.129 | 0.025 | 0.103 | 0.0081 | 0.077 | 0.0038 | 1.105 | 0.0260 |

Supplemental Table 3. Cognitive task performance scores (mean plus SEM) on Day 1 and Day 56. In this instance the cognitive tasks were used simply to elicit metabolic and CBF responses and no pre-treatment baseline measure was taken. N = the number of complete datasets included in the analysis. *, p < 0.01 in comparison to placebo on that day.

|  |  |  | Day 1 | | Day 56 | |
| --- | --- | --- | --- | --- | --- | --- |
| Task period | Treatment | N | Mean | SEM | Mean | SEM |
| Serial 3s total  (number) | Placebo | 27 | 87.8 | 4.9 | 88.1 | 4.9 |
|  | 1RDA+Q10 | 30 | 94.8 | 6.0 | 94.3 | 6.0 |
|  | 3RDA | 28 | 80.1 | 5.0 | 81.8 | 4.9 |
| Serial 3s error  (number.) | Placebo | 27 | 5.3 | 0.7 | 5.8 | 0.9 |
|  | 1RDA+Q10 | 30 | 6.0 | 0.7 | 5.3 | 0.8 |
|  | 3RDA | 28 | 5.8 | 0.8 | 5.1 | 0.6 |
| Serial 7s total  (number) | Placebo | 27 | 59.2 | 3.9 | 58.4 | 4.4 |
|  | 1RDA+Q10 | 30 | 60.8 | 4.2 | 62.3 | 4.9 |
|  | 3RDA | 28 | 53.7 | 3.2 | 55.8 | 3.3 |
| Serial 7s error  (number.) | Placebo | 27 | 6.2 | 0.9 | 4.8 | 0.9 |
|  | 1RDA+Q10 | 30 | 6.2 | 0.8 | 7.2 | 1.1 |
|  | 3RDA | 28 | 5.9 | 0.9 | 6.4 | 0.8 |
| Serial 17s total  (number) | Placebo | 27 | 37.6 | 2.6 | 37.9 | 2.8 |
|  | 1RDA+Q10 | 30 | 40.7 | 3.0 | 40.1 | 2.9 |
|  | 3RDA | 28 | 34.3 | 2.2 | 35.5 | 2.1 |
| Serial 17s error  (number.) | Placebo | 27 | 5.2 | 0.7 | 5.4 | 0.8 |
|  | 1RDA+Q10 | 30 | 5.6 | 0.8 | 7.1 | 0.9 |
|  | 3RDA | 28 | 5.9 | 0.8 | 4.8 | 0.7 |
| Stroop correct  (%) | Placebo | 27 | 97.9 | 0.4 | 97.8 | 0.3 |
|  | 1RDA+Q10 | 29 | 97.6 | 0.5 | 97.8 | 0.5 |
|  | 3RDA | 26 | 98.1 | 0.3 | 98.4 | 0.3 |
| Stroop reaction  time  (msecs) | Placebo | 27 | 674.5 | 11.0 | 672.0 | 13.0 |
|  | 1RDA+Q10 | 29 | 656.7 | 14.3 | 674.2 | 16.2 |
|  | 3RDA | 26 | 670.5 | 8.6 | 678.9 | 11.9 |
| 3-Back correct  (%) | Placebo | 26 | 86.4 | 1.2 | 87.0 | 1.5 |
|  | 1RDA+Q10 | 28 | 82.4* | 1.7 | 81.1* | 1.6 |
|  | 3RDA | 26 | 84.2 | 1.4 | 85.4 | 1.5 |
| 3-Back reaction  Time  (msec) | Placebo | 26 | 822.1 | 44.9 | 812.1 | 41.4 |
|  | 1RDA+Q10 | 28 | 823.3 | 34.2 | 769.1 | 29.3 |
|  | 3RDA | 26 | 880.6 | 41.4 | 867.0 | 34.4 |

Supplemental Table 4. Mean subjective difficulty and mental fatigue data (+SEM) from Day 1 and Day 56.

|  |  |  | Day 1 | | | |  | Day 56 | | | |
| --- | --- | --- | --- | --- | --- | --- | --- | --- | --- | --- | --- |
|  |  |  | Difficulty | | Fatigue | |  | Difficulty | | Fatigue | |
|  | Treatment | N | Mean | SEM | Mean | SEM | N | Mean | SEM | Mean | SEM |
| Control task | 1RDA+CoQ10 | 27 | 24.22 | 2.64 | 43.89 | 3.40 | 28 | 24.04 | 2.58 | 43.39 | 3.06 |
|  | 3RDA | 28 | 21.61 | 2.59 | 47.96 | 3.34 | 23 | 20.78 | 2.85 | 48.39 | 3.38 |
|  | Placebo | 28 | 25.68 | 2.59 | 45.14 | 3.34 | 25 | 28.68 | 2.73 | 49.08 | 3.24 |
| Stroop task | 1RDA+CoQ10 | 27 | 38.00 | 3.04 | 49.89 | 3.30 | 28 | 36.11 | 2.90 | 49.11 | 3.13 |
|  | 3RDA | 28 | 26.61 | 2.98 | 47.14 | 3.24 | 23 | 26.96 | 3.20 | 47.83 | 3.45 |
|  | Placebo | 28 | 37.29 | 2.98 | 49.82 | 3.24 | 25 | 31.64 | 3.07 | 49.04 | 3.31 |
| Serial 3s | 1RDA+CoQ10 | 27 | 45.07 | 3.26 | 52.59 | 3.16 | 28 | 41.21 | 3.24 | 49.29 | 3.15 |
|  | 3RDA | 28 | 41.68 | 3.20 | 57.57 | 3.10 | 23 | 42.57 | 3.58 | 48.70 | 3.48 |
|  | Placebo | 28 | 43.71 | 3.20 | 54.29 | 3.10 | 25 | 40.16 | 3.43 | 51.08 | 3.34 |
| Serial 7s | 1RDA+CoQ10 | 27 | 56.74 | 3.01 | 55.30 | 2.94 | 28 | 52.93 | 3.07 | 55.93 | 3.26 |
|  | 3RDA | 28 | 54.04 | 2.95 | 55.36 | 2.88 | 23 | 54.87 | 3.39 | 53.17 | 3.60 |
|  | Placebo | 28 | 55.32 | 2.95 | 56.04 | 2.88 | 25 | 48.12 | 3.25 | 51.76 | 3.45 |
| Serial 17s | 1RDA+CoQ10 | 27 | 64.19 | 2.89 | 52.22 | 3.33 | 28 | 62.89 | 2.90 | 52.00 | 3.31 |
|  | 3RDA | 28 | 61.93 | 2.84 | 57.18 | 3.27 | 23 | 59.78 | 3.20 | 51.17 | 3.65 |
|  | Placebo | 28 | 60.64 | 2.84 | 52.57 | 3.27 | 25 | 62.72 | 3.07 | 54.48 | 3.50 |
| 3-Back | 1RDA+CoQ10 | 27 | 64.96 | 3.05 | 56.81 | 3.16 | 28 | 68.00 | 3.05 | 58.71 | 3.28 |
|  | 3RDA | 28 | 63.39 | 2.99 | 54.93 | 3.10 | 23 | 63.70 | 3.37 | 50.43 | 3.61 |
|  | Placebo | 28 | 61.89 | 2.99 | 56.32 | 3.10 | 25 | 53.88 | 3.23 | 55.44 | 3.47 |
